# Supplementary material for: A Computational Methodology to Screen Activities of Enzyme Variants
Source: PLoS One. 2012 Dec 17;7(12):e49849. doi: 10.1371/journal.pone.0049849 (PMC3524253; doi:10.1371/journal.pone.0049849)
Supplement: Text S1 — Difference MOZYME/PM6 energies; Amino acid sequences in models (a), (b) and (c); Transition state verification animation and URL of repository for modeling scripts. (PDF) [file pone.0049849.s001.pdf]

S1 A Computational Methodology to Screen Activities of Enzyme Variants: Supplementary Material

Martin Hediger, Luca De Vico, Allan Svendsen, Werner Besenmatter, Jan H. Jensen. Corresponding Author, Email: jhjensen@chem.ku.dk

Difference MOZYME/PM6 Energies

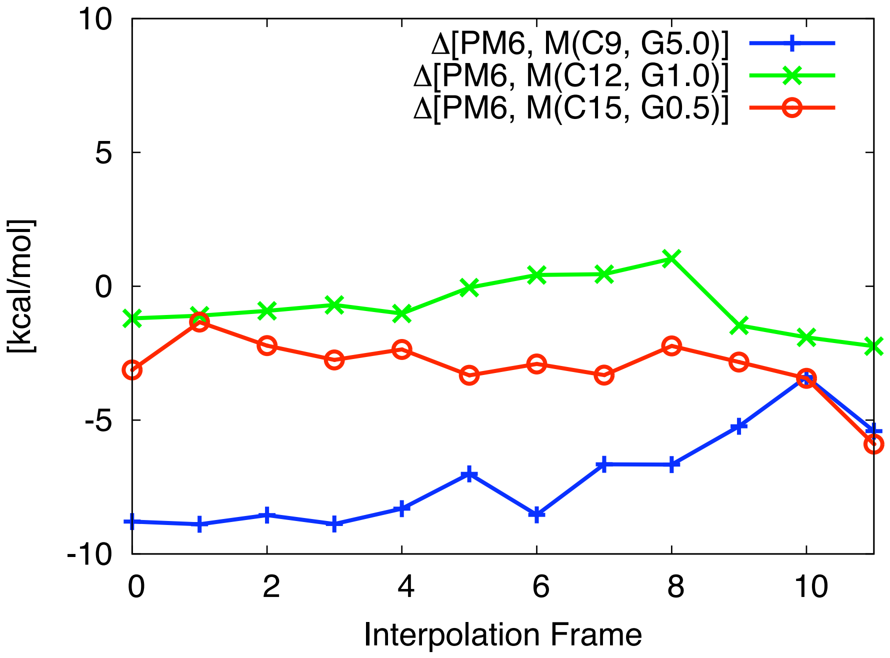

Figure 15. Difference ΔH_f^{PM6} - ΔH_f^{MOZYME} between PM6 and MOZYME (not reorthogonalized) energy at each interpolation frame in (**b**). Average difference (in kcal/mol): -7.19, -0.73, -2.98 for NDDO cutoff 9, 12, and 15Å, respectively.

Amino Acids in Models (a), (b) and (c)

All amino acids in the indicated ranges included in the respective models.

(**a**): 38-42, 103-106, 134, 187, 189, 223-225, 278, 281

(**b**): 37-50, 102-106, 131-135, 139-141, 156-158, 186-192, 220-226, 277-287

(**c**): 35-52, 66-83, 101-114, 128-141, 153-158, 163-167, 172-174, 179-193, 200-204, 223-233, 277-285

Transition State Verification

The animation of the vibration can be found online under:

`http://www.youtube.com/watch?v=7ZLaqH2xDy8`

Git Repository for Scripts

The scripts used in the development of this method are available under:

`git@github.com:mzhKU/Enzyme-Screening.git`
